# Fusobacteria modulate oral carcinogenesis and promote cancer progression

**DOI:** 10.1080/20002297.2020.1849493

**Published:** 2020-11-30

**Authors:** Amani M. Harrandah, Sasanka S. Chukkapalli, Indraneel Bhattacharyya, Ann Progulske-Fox, Edward K. L. Chan

**Affiliations:** a Department of Oral Biology, University of Florida College of Dentistry, Gainesville, Florida, USA; b Department of Oral Biology, Umm AlQura University, Makkah, Saudi Arabia; c Center for Molecular Microbiology, University of Florida College of Dentistry, Gainesville, Florida, USA; d Department of Oral & Maxillofacial Pathology, University of Florida College of Dentistry, Gainesville, Florida, USA

**Keywords:** 4NQO, oral cancer, periodontal bacteria, inflammation, *fusobacterium nucleatum*

## Abstract

**Background:** Evidence suggest periodontal bacterial infection can contribute to oral cancer initiation and progression.

**Aim:** To investigate the effects of periodontal bacteria on oral cancer cell behavior using a cell-based system and a mouse carcinogenesis model.

**Methods:** Oral cancer cell lines were polyinfected with four periodontal bacteria. Cytokine levels and relative changes in oncogene mRNA expression were determined post-infection. Oral tumours in mice induced by 4-nitroquinoline-1-oxide (4NQO) were compared with and without administrating periodontal bacteria.

**Results:** Polyinfected oral cancer cells had upregulated MMP1, MMP9, and IL-8. The expression of cell survival markers MYC, JAK1, and STAT3 and epithelial-mesenchymal transition markers ZEB1 and TGF-β were also significantly elevated. Monoinfections showed F. nucleatum alone had comparable or greater effects than the four bacteria together. Fusobacterial culture supernatant, primarily LPS, was sufficient to induce IL-8 secretion, demonstrating that direct contact of live Fusobacteria with cancer cells might not be required to exert changes in cancer cell behaviour. In the 4NQO-induced oral tumour model, mice infected with bacteria developed significantly larger and more numerous lesions compared to those not infected.

**Conclusion:** This study demonstrated that Fusobacteria could potentially enhance cancer cell invasiveness, survival, and EMT when presented in the oral tumour microenvironment.

**Abbreviations**: 4NQO, 4-nitroquinoline-1-oxide; ELISA, enzyme-linked immunosorbent assay; EMT, epithelial–mesenchymal transition; IL-8, interleukin-8; JAK1, Janus kinase 1; LPS, lipopolysaccharide; MMP, matrix metalloproteinase; OSCCs, oral squamous cell carcinomas; PK, proteinase K; PMB, Polymyxin B; qRT-PCR, quantitative real-time polymerase chain reaction; STAT3, signal transducer and activator of transcription 3; TGF-β, transforming growth factor beta; ZEB1, zinc finger E-Box binding homeobox 1

## Introduction

Oral cancer is one of the most common cancers that affect public health [1]. In the USA alone, 53,260 patients will be diagnosed with oral cavity and pharyngeal cancers and more than 10,000 people will die from oral cancer in 2020 [2]. Oral squamous cell carcinomas (OSCCs) are the most common oral cancers that affect the oral epithelium. The survival rate and the quality of life of patients with OSCCs are highly dependent on early diagnosis and management [3]. Data in the literature support the link between inflammation and cancer progression. For example, there is increased risk of gastric cancer and colon cancer in patients with gastritis and ulcerative colitis, respectively [4]. In addition, about 15% of cancers may be triggered by infections, with recent evidence suggesting that microorganisms can promote cancer progression by inducing inflammation [5]. Since periodontal disease is associated with chronic inflammation [6], oral pathogens may have an important role in oral cancer initiation and progression [7]. Several studies have suggested an association between chronic periodontitis and increased risk of oral cancer [8–10]. A recent meta-analysis showed a 2-fold increase in the risk of oral cancer in patients with periodontal disease [11]. Additionally, some cancer patients suffer from depression along with the side effects of chemotherapy/radiotherapy, all of which can hinder them from maintaining proper oral hygiene. This can result in increased gingival bleeding and incidence of periodontal infection in these patients [12].

Biofilms on OSCCs are also reported to be rich with anaerobic bacteria associated with periodontal disease [13,14]. Adding to that, there is an association between poor oral hygiene and oral cancer [15]. In fact, many studies have reported the deleterious effect of periodontal pathogens on different types of cancer including oral cancer [7]. For example, periodontal pathogen *F. nucleatum* was reported to induce chemoresistance in colorectal cancer cells by upregulating autophagy [16]. Moreover, recent study on colorectal cancer have shown that the FAD-A protein from *F. nucleatum* upregulate Annexin A1 on cancer cells through E‐cadherin and this promotes carcinogenesis [17]. Also, another periodontal pathogen *P. gingivalis* has been reported to induce oral cancer cells invasiveness by inducing IL-8 secretion and MMPs upregulation [18]. However, there is a gap in the understanding of the biological effects of periodontal bacteria on oral cancer cell behaviour. Hence, this study was designed to investigate the effects of periodontal pathogens on cultured oral cancer cells in vitro and follow up with testing whether selected oral bacteria could enhance the formation of precancerous lesion in a mouse oral carcinogenesis model.

## Materials and methods

### Cell culture

HN and BHY cells were head and neck cancer cells purchased from Deutsche Sammlung von Mikroorganismen und Zellkulturen, GmbH (DSMZ, Braunschweig, Germany). OQ01 was a primary cultured head and neck cancer cell line provided by Dr. Lung-Ji Chang, University of Florida [19]. These cancer cell lines were maintained in DMEM/F12 supplemented with 10% fetal bovine serum, 100 µg/ml streptomycin, and 100 units/ml penicillin in a humidified chamber (37°C, 5% CO_2_) as previously described [20,21].

### Bacterial culture

*P. gingivalis* strain ATCC 53,977, *F. nucleatum vincentii* ATCC 49,256, *F. nucleatum polymorphum* ATCC 10,953, *F. periodonticum* ATCC 33,693, *Treponema denticola* ATCC 35,404 and *Tannerella forsythia* ATCC 43,037 were grown as previously described at 37°C in an anaerobic chamber [22]. Bacteria were grown in their respective culture media until their logarithmic growth phase, with growth rate measured by optical density at 550 nm. Culture characteristics and gram stains were assessed for all cultures prepared in this study. Culture supernatants were collected after centrifugation at 12,000 × g for 10 min and used for experiments described below.

### Infection of cancer cells

Cancer cells were seeded at 5 x 10^5^/ml in wells of 6-well plates at 37°C overnight. A combination of four live bacteria (*P. gingivalis, F. nucleatum vincentii, T. denticola*, and *T. forsythia* at 1:1:1:1 ratio) or individual bacteria were added at a multiplicity of infection (MOI) of 100 for each bacterium. As controls, oral cancer cells were also cultured in medium alone. Plates were incubated at 37°C with 5% CO_2_ for 6 or 24 h. Supernatants and cell lysates were collected for cytokine analyses and RNA isolation.

### Enzyme-linked immunosorbent assay (ELISA)

Secreted cytokines and matrix metalloproteinases (MMPs) in culture supernatants were measured by ELISA using an Opt EIA ELISA kit as recommended by ELISA kit manufacturers (BD Biosciences and Affymetrix eBioscience). Absorbance was measured using a microplate reader (Model 680, Bio-Rad) and converted into concentration using standard curves of respective purified recombinant human proteins.

### mRNA expression

Total RNA from harvested cells was isolated using the mirVana miRNA Isolation Kit (Thermo Fisher) and RNA concentrations were determined using a NanoDrop ND-1000 spectrophotometer. For mRNA (STAT3, JAK1, MYC, MMP1, MMP9, and ZEB1) qRT-PCR analysis, fold changes in expression levels were calculated after normalization to 18S RNA as previously described [19,20,23].

### Cell invasion assay

Cell invasion assays were carried out using Transwell chambers (8 µm pore size; Corning) [20]. Cancer cells (5 x 10^4^) infected with bacteria for 4 h or mock-infected control cells were suspended in 200 μl serum-free DMEM medium in 1:10-diluted matrigel-coated upper chamber (Matrigel, BD Biosciences, San Jose, CA). DMEM containing 10% FBS was added in the lower chamber. After 24 h, cells migrated to the bottom of the filter were fixed, stained with 0.05% crystal violet in 20% methanol for 5 min, and counted in 5 fields/filter at 200x magnification [20].

### Characterization of the active component in *F. periodonticum* supernatant

The following treatments were performed on the *F. periodonticum* bacterial culture supernatants to examine their ability to induce IL-8 in OQ01 cells:

*Proteinase K treatment. F. periodonticum* bacterial culture supernatant 100 µl was incubated with Proteinase K (Thermo Fisher) at 0, 2, or 20 ug/ml for 5 min at 55°C. Next, to deactivate Proteinase K, the mixture was heated at 95°C for 10 min. The supernatant with deactivated enzyme was then incubated with cancer cells for 24 h. Supernatant treated similarly but without the addition of Proteinase K and uninfected cells were used as additional controls. OQ01 cell supernatants were analyzed for IL-8 production by ELISA.

*Nucleic acid depletion. F. periodonticum* bacterial supernatant 100 µl was incubated with 10 µl of 200 K units/ml micrococcal nuclease (Cell Signaling) for 20 min at 37°C. To deactivate the nuclease, the mixture was heated at 65°C for 10 min. The supernatant with deactivated enzyme was incubated with cancer cells for 24 h. The efficiency of nucleic acid depletion using micrococcal nuclease was confirmed by agarose gel electrophoresis. OQ01 cell supernatants were analyzed for IL-8 production by ELISA.

*LPS depletion*. To determine whether polymyxin B (PMB) could effectively deplete LPS, *Salmonella enterica* LPS (Sigma-Aldrich) was used as a control to stimulate OQ01 cells to induce IL-8 production. Next, 100 units/ml PMB was mixed with *Salmonella enterica* LPS to determine its efficiency in depletion and inhibition of IL-8 production. *F. periodonticum* bacterial supernatant 100 µl was mixed similarly with PMB and the PMB/supernatant mixture was added to OQ01 cells for 24 h. Untreated *F. periodonticum* bacterial supernatants were used as positive controls, while uninfected cells and polymyxin B alone were used as negative controls. OQ01 cell supernatants were analyzed for IL-8 production by ELISA.

### Western blot

Oral cancer cell lysates were analyzed on 10% polyacrylamide gels and transferred to nitrocellulose membranes. The dilutions of primary antibodies were 1:1000 for rabbit anti-ZEB1 (Cell Signaling) and 1:200 for mouse anti-actin antibodies (Sigma-Aldrich). Secondary antibodies conjugated to horseradish peroxidase were used at 1:10,000 dilutions (Southern Biotech).

### 4-nitroquinoline-1-oxide (4NQO) carcinogenesis model

Eight-weeks-old C57BL/6 J male mice were obtained from The Jackson Laboratory and housed in microisolator cages in specific pathogen-free environment. Mice were randomly divided into three groups (6 per group). Group I was untreated and served as a sham-control. Group II and III were administered 50 µg/ml 4NQO (Sigma) for 8 weeks in their drinking water from week 4 to 12 (see Figure 5A). Group III was repeatedly infected (3 times per week) with *P. gingivalis* (strain 53977) for the first 2 weeks, *F. nucleatum* (strain 49,256) from week 2 to 4, and a mixture of both from week 12 to 18. Each inoculation mixture consisted of 100 μl of 10^9^ cfu/ml bacteria suspended in 2% carboxymethylcellulose as described [22]. The sequence of infection of *P. gingivalis* and *F. nucleatum* was done to simulate the natural sequence in the colonization of oral bacteria in the oral cavity. During the period of 4NQO administration, mice were not infected with the bacteria mixture. All mice were sacrificed in week 18 and their tongues were harvested, immersion-fixed in 4% paraformaldehyde, and processed and embedded in paraffin. Lesion area was calculated from the macroscopic lesion using Axiovison software (Carl Zeiss, Germany). Sections of 5 µm thickness were stained with H&E and histopathologically scored in a blinded fashion by an expert oral pathologist (I.B.). Animal experiments were approved by the Institutional Animal Care and Use Committee, University of Florida (approval 20,170,012). The University of Florida strictly follows U.S. Public Health Services policy, the Animal Welfare Act and Animal Welfare Regulations, and the guide for the Care and Use of Laboratory Animals.

### Statistical analysis

All expression results are presented as mean±SEM from three independent experiments. Comparisons of treated to control were analyzed using two-tailed Student’s test and multiple independent groups were analyzed using one-way ANOVA performed using Prism (Graph Pad Software).

## Results

*Enhanced expression of STAT3, JAK1, MYC and EMT markers in* F. nucleatum*-infected oral cancer cell lines*

STAT3, JAK1, and MYC are three oncogenes that have both anti-apoptotic and proliferative effects on cancer cells [24]. Therefore, it is expected for these oncogenes to be upregulated in cancer cells compared to normal cells. In this study, we aim to investigate further upregulation of these oncogenes by periodontal bacteria and subsequently enhancing carcinogenesis.

**Figure 1.**
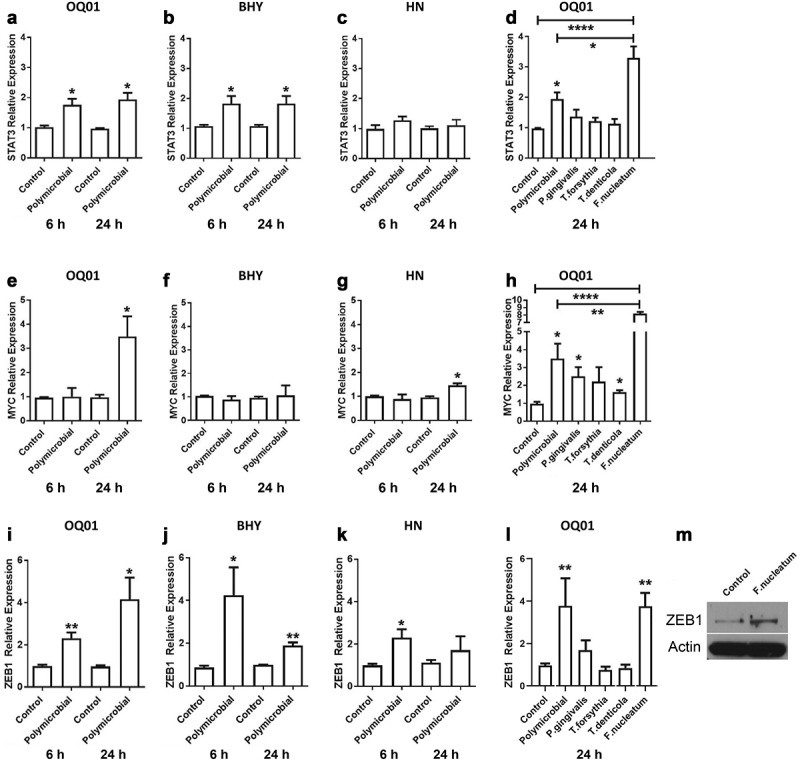
*F. nucleatum* induces the expression of STAT3, MYC, and ZEB1 in oral cancer cells *in vitro*. (A-C) Elevated STAT3 mRNA expression levels after infection with four periodontal bacteria combined (polymicrobial infection) in oral cancer cell lines OQ01, BHY, and HN for 6 and 24 h. (D) The effect of single bacterial infection on the expression level of STAT3 in OQ01 cells after 24 h of infection. (E-G) Significantly elevated MYC mRNA expression level in OQ01 and HN, but not BHY, after 24 h of polymicrobial infection. (H) Comparison of the effects of single bacterial infections and polymicrobial infection on MYC mRNA expression in OQ01 cells after 24 h of infection. (I-K) Increased ZEB1 mRNA expression after polymicrobial infection of three oral cancer cell lines for 6 and 24 h. (L) Increased ZEB1 expression in OQ01 cells after single bacterial infection with *F. nucleatum*. (M) Western blot analysis of ZEB1 protein levels 24 h after *F. nucleatum* infection in OQ01 cells compared to uninfected cells. Uninfected cells were used as controls in all experiments. All mRNA levels were determined using qRT-PCR. All results are presented as mean ± SEM from three independent experiments. *, P <.05; **, P <.005; ****, P <.0001

Infection with *P. gingivalis* could activate JAK/STAT3 pathway in normal gingival epithelial cells [25], While *F. nucleatum* could upregulate MYC in colon cancer cells through E-cadherin pathway [26]. Therefore, the change in expression for these oncogenes was analyzed after bacterial infection of oral cancer cells. To ensure that the effect observed from bacterial infection was not specific to a single cell line, three different oral cancer cell lines were examined. Accordingly, we measured the expression of STAT3, JAK1, and MYC in three oral cancer cell lines (OQ01, HN, BHY) after polymicrobial infection with the combination of *P. gingivalis, F. nucleatum, T. denticola*, and *T. forsythia* by qRT-PCR. STAT3 expression was enhanced almost two-fold in infected OQ01 and BHY cells compared to untreated controls Figure 1(A,B) and slightly increased in HN cells Figure 1C, after both 6 and 24 h of polymicrobial infection. Enhanced JAK1 expression was also seen in OQ01 and BHY 6 and 24 h post-infection (Appendix Fig. 1A,B). The same trend was seen in HN although the differences were not statistically significant (Appendix Fig. 1C). In contrast, enhanced MYC expression was detected in OQ01 and HN cells Figure 1(E,G), but not in BHY Figure 1F, and only at 24 h post-infection. In this pilot study, the activation states of the oncogenes was not examined. However, mRNA level of STAT3 was reported consistent with protein level in gingival epithelial cells [27] and MYC mRNA and protein level was found closely related in colon cancer cells after *Fusobacterial* infection [26]. Follow up studies will be required further define whether there would be changes in protein status including post-translational modifications such as phosphorylation.

Next, we investigated whether these individual bacterial species alone could induce expression of these oncogenes. Single bacterial infections of OQ01 cells revealed that *F. nucleatum* alone led to a significant increase in STAT3 Figure 1D and MYC expression Figure 1H compared to control as well as to the polymicrobial infection. However, increased JAK1 expression appeared to be a synergistic effect from the four bacteria combined (Appendix Fig. 1D).

Cancer cells undergo epithelial-mesenchymal transition (EMT) to initiate metastasis [28,29]. Increased expression of ZEB1 and TGF-β is a hallmark of EMT [30]. The same polymicrobial infection led to increased ZEB1 mRNA levels in all three oral cancer cell lines in both 6 and 24 h post-infection Figure 1(I-K). Simultaneously, there was an increase in TGF-β secretion in the three cell lines after polybacterial infection (Appendix Fig. 2A-C). *F. nucleatum* alone also significantly induced ZEB1 Figure 1L and TGF-β (Appendix Fig. 2D) expression. To confirm the effect of *F. nucleatum* on ZEB1 expression, Western blot analysis showed increased ZEB1 protein after 24 h *F. nucleatum* infection Figure 1M.

F. nucleatum *enhanced MMP1, MMP9, and IL-8 expression and cancer cell invasiveness*

**Figure 2.**
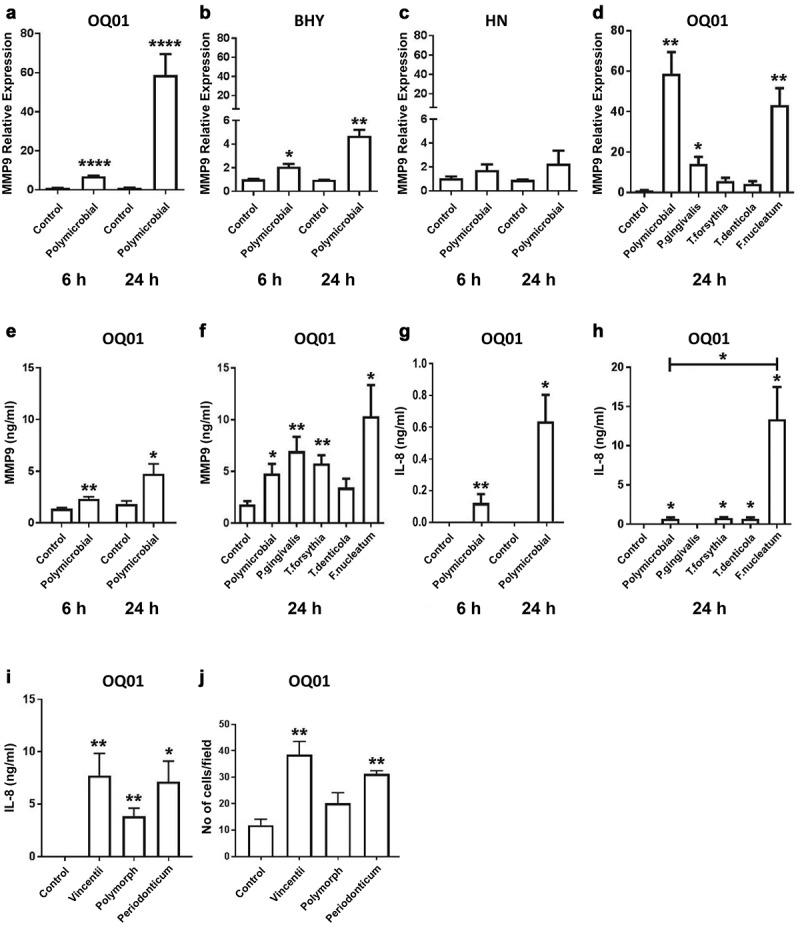
*F. nucleatum* enhances MMP9 and IL-8 expression and cancer cell invasiveness *in vitro*. (A-C) Elevated MMP9 mRNA expression in OQ01 and BHY, but not HN, after polymicrobial infection for 6 and 24 h. (D) Increased MMP9 expression in OQ01 cells after single infection with *P. gingivalis* or *F. nucleatum*. (E) Increased MMP9 secretion in OQ01 cell culture supernatant after polymicrobial infection for 6 and 24 h. (F) Comparison of the effects of single bacterial infections and polymicrobial infection on MMP9 secretion in OQ01 cell supernatant. (G) Elevated IL-8 secretion in OQ01 cell supernatant after polymicrobial infection for 6 and 24 h. (H) Comparison of the effects of single bacterial infections and polymicrobial infection on IL-8 secretion in OQ01 cell supernatant. (I) elevated IL-8 levels in OQ01 cell supernatant after infection with three different *Fusobacteria* strains. (J) *In vitro* invasion assay was performed using Matrigel-coated Transwell filters. Cells infected with three *Fusobacteria* strains were incubated for 24 h on the filter. Cells on the upper surface were removed and cells on the lower surface were fixed and stained. Five fields at 200x magnification were counted per each filter. All mRNA levels were determined using qRT-PCR. IL-8 and MMP9 protein levels in culture supernatants were determined by ELISA. Uninfected cells were used as controls. All results are presented as mean ± SEM from three independent experiments. *, P <.05, **, P <.005, ***, P <.0005, ****, P <.0001

Periodontal bacteria are known to induce MMPs expression in periodontal tissues and enhance tissue destruction during periodontal disease progression [31]. Hence, our next step was to examine the effect of periodontal bacteria on the expression of MMPs in oral cancer cells. Enhanced MMP9 expression was observed in OQ01 and BHY Figure 2(A,B) and slightly increased in HN cells after infection Figure 2C. We also measured increased MMP9 protein secretion in culture supernatants of OQ01 cells after polybacterial infection by ELISA Figure 2E. Similarly, MMP1 mRNA expression was also enhanced in all cell lines tested 24 h after polymicrobial infection (Appendix Fig. 3A-C). Furthermore, exposure of OQ01 cells to the polymicrobial infection resulted in significantly increased IL-8 secretion compared to untreated cells Figure 2G.

When single bacterial infections in OQ01 cells were examined, *F. nucleatum* appeared to have the most effect among the four bacteria in upregulating MMP1 (Appendix Fig. 3D) and MMP9 Figure 2(D,F). *P. gingivalis* alone also showed elevated MMP9 level compared to control Figure 2D. A dramatic increase in IL-8 secretion was also observed with *F. nucleatum* alone compared to control and to polymicrobial infection Figure 2H. There was no substantial difference between three different *Fusobacteria* strains (*F. nucleatum vincentii, F. nucleatum polymorphum*, and *F. periodonticum*) in enhancing IL-8 expression Figure 2I.

We then performed an *in vitro* invasion assay to measure the effects of different strains of *Fusobacteria* on cancer cell invasiveness. Infection with any of the three *Fusobacteria* strains enhanced OQ01 cell invasiveness *in vitro*, although no statistically significant difference was observed with *F. polymorphum* Figure 2J.

### Bacterial culture supernatant alone induced expression of IL-8 and MMPs

The next experiments were designed to address the mechanism by which *Fusobacteria* can induce this invasive phenotype in oral cancer cells. To determine if live bacteria are needed to induce this phenotype, we performed experiments comparing live versus heat-killed bacteria and bacterial culture supernatant of *F. periodonticum*.

**Figure 3.**
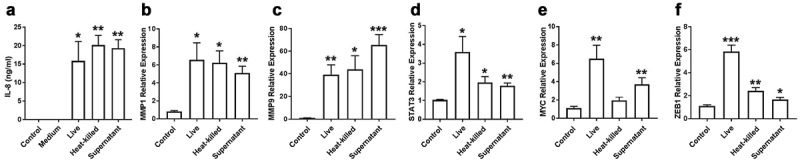
*F. periodonticum* culture supernatant alone is sufficient to induce IL-8 secretion and MMPs expression, while live bacteria give the strongest enhanced expression of STAT3, MYC, and ZEB1 in OQ01 oral cancer cells. (A) Comparison of the effects of live bacteria, heat-killed bacteria, and bacterial culture supernatant on IL-8 secretion in OQ01 cells after 24 h of incubation. Untreated cells (Control) and cells treated with bacterial culture medium alone (Medium) were negative controls. IL-8 protein level was determined by ELISA. (B-F) MMP1, MMP9, STAT3, MYC, and ZEB1 mRNA expression after treatment with live bacteria, heat-killed bacteria, or bacterial culture supernatant were determined by RT-PCR. All supernatants were obtained from *F. periodonticum*. Uninfected cells were used as controls. All results are presented as mean ± SEM from three independent experiments. *, P <.05, **, P <.005, ***, P <.0005

Our results showed that bacterial supernatant alone was sufficient to induce IL-8 secretion in OQ01 cells at levels comparable to cells treated with live or heat-killed bacteria Figure 3A. We focused on IL-8 secretion because it has been reported that IL-8 can enhance tumour invasion and elevated expression has been associated with poor patient prognosis [18,32]. Similarly, bacterial supernatant alone was able to enhance the expression of MMP1 and MMP9 in the same cells Figure 3(B,C). In contrast, live bacteria induced increased expression of STAT3, MYC, and ZEB1 compared to heat-killed bacteria and culture supernatant Figure 3(D-F). Comparable results were observed with other *Fusobacteria* strains (data not shown).

**Figure 4.**
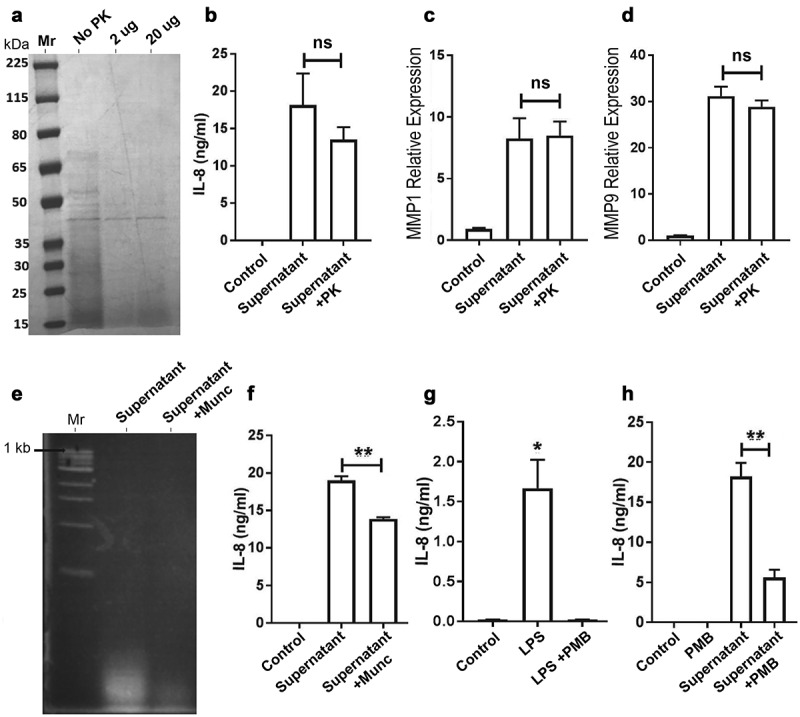
Effects of protein and nucleic acid digestions as well as functional LPS depletion on IL-8 secretion in OQ01 cells. (A) Polyacrylamide gel electrophoresis and Coomassie Blue stain analysis of *F. periodonticum* bacterial culture supernatant before and after treatment with two different concentrations of proteinase K (PK) showing almost complete degradation of proteins in the supernatant. (B) Comparing the effects of untreated (Control), supernatant, and PK-treated supernatant on IL-8 secretion from OQ01 cells after 24 h of incubation. Similarly, proteinase K treatment of supernatant did not affect the expression of MMP1 (C) and MMP9 (D) in OQ01 cells after 24 h of incubation. (E) Agarose gel and ethidium bromide staining analysis of bacterial culture supernatant before and after treatment with micrococcal nuclease (Mnuc) showing essentially complete degradation of nucleic acids in the supernatant. (F) Comparison of the effects of untreated, supernatant, and Mnuc-treated bacterial supernatant on IL-8 secretion in OQ01 cells after 24 h of infection. (G) Comparison of the effects of *Salmonella enterica* LPS alone and LPS+PMB treatment on IL-8 secretion in OQ01 cells after 24 h of incubation. (H) Comparison of the effects of untreated, PMB alone, supernatant, and PMB-treated bacterial supernatant on IL-8 secretion in OQ01 cells after 24 h of infection. All IL-8 protein levels were determined by ELISA. Uninfected cells were used as controls. All results are presented as mean ± SEM from three independent experiments. ns, not significant; *, P <.05; **, P <.005

To characterize the bacterial culture supernatant component responsible for the effect exhibited in oral cancer cells, we examined whether the putative active factor(s) in the supernatant was a polypeptide, which would be highly sensitive to Proteinase K digestion. As expected, untreated supernatant showed a complex mixture of polypeptides with molecular masses ranging from >65kDa to small peptides of <15kDa (Figure 4A, No PK). Digestion with 2 or 20 ug Proteinase K showed substantial reduction in intensity of the majority of polypeptides. The Proteinase K-treated and boiled supernatant (Supernatant+PK) was added to OQ01 cells for 24 h and compared to control supernatant without Proteinase K treatment (Supernatant, Fig. 4B). Although there was ~20% decrease in IL-8 secretion, protein digestion and boiling did not have a major effect on IL-8 secretion in OQ01 cells. Similarly, there was no significant effect on MMP1 and MMP9 expression Figure 4(C, D). This indicated that polypeptides were not the main factors in the culture supernatant responsible for inducing the IL-8 secretion and MMP expression observed in OQ01 cells.

Next, we examined the effect of nucleic acid depletion of the supernatant on IL-8 secretion in OQ01 cells. Nucleic acid depletion using micrococcal nuclease (Mnuc) was effective in a control system as demonstrated by agarose gel electrophoresis Figure 4E and yet it had only a minor effect (20% decrease) on IL-8 secretion from OQ01 cells Figure 4F.

To investigate whether the active factor was LPS, polymyxin B (PMB) was employed to bind and inactivate LPS from the supernatant. First, purified LPS from *Salmonella enterica* added to OQ01 cells induced IL-8 secretion, which was completely abolished with the addition of PMB Figure 4G. Addition of PMB to the bacterial culture supernatant abolished 75% of IL-8 secretion Figure 4H, indicating that LPS was a major component of bacterial supernatant responsible for enhancing IL-8 secretion. Note that the same bacterial supernatant treated with PMB did not result in reduced MMPs expression (data not shown) indicating that LPS was not primarily responsible for elevated MMPs expression in OQ01 cells. Altogether, it appears that the interaction between *Fusobacteria* and these oral cancer cells is not solely through LPS/TLR4 pathway. Furthermore, this interaction might occur through multiple biological pathways resulting in upregulation of multiple oncogenes.

### P. gingivalis and F. nucleatum promote tumour progression in vivo

**Figure 5.**
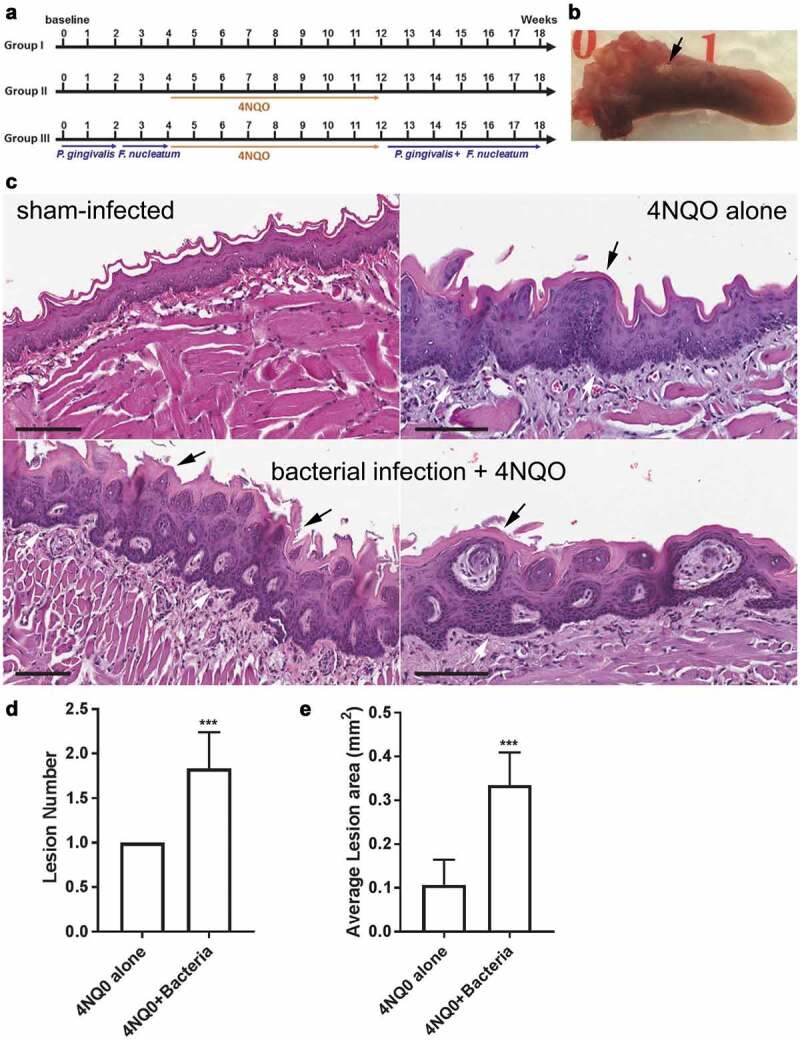
*P. gingivalis* and *F. nucleatum* promote oral cancer progression *in vivo*. (A) Schematic summary of the periodontal pathogen-associated oral tumorigenesis model. Mice in Groups II and III were administered oral carcinogen 4NQO in their drinking water from week 4 to 12. In Group III, chronic periodontitis was induced by repeated oral infection with *P. gingivalis* alone for 2 weeks, *F. nucleatum* alone for another 2 weeks, and a mixture of *P. gingivalis* and *F. nucleatum* from week 12 to 18. (B) A representative image of tongue tissue showing an oral lesion (arrow) from a mouse in Group III. (C) H&E-stained section of sham-infected (Group I) mouse tongue displaying normal rete ridge formation with well-organized basal layer (sham-infected, top left). H&E-stained section of tongue from a mouse in Group II showing significant hyperkeratosis (black arrow, top right, 4NQO alone), hyperchromatism and mild hyperplasia (white arrows). H&E-stained section of tongue from a mouse in Group III showing significant hyperkeratosis (black arrows), hyperchromatism, hyperplasia, and increased nuclear/cytoplasmic ratios (white arrows, bacterial infection + 4NQO, lower panels). Higher magnification (200x) of tongue from another mouse in Group III again showing hyperkeratotic surface with foci of basilar hyperplasia nuclear hyperchromatism (white arrows, lower right). (D) The average number of macroscopic lesions differences between Group II and III. (E) The average lesion area in Group II and III. All data are presented as mean ± SEM, n = 6 mice per condition. Scale bars, 100 µm

Data from our *in vitro* experiments suggested that periodontal bacteria can enhance cancer progression. To investigate if these effects also occur *in vivo*, we used the 4NQO-induced oral carcinoma mouse model because it simulates human OSCC carcinogenesis progression over a reasonably short period of time in immunocompetent mice [33,34]. Although *F. nucleatum* showed most dramatic effect on oncogene upregulation and IL-8 secretion, *P. gingivalis* had an effect on MYC, MMP1 and MMP9. Moreover, it has been reported that combined oral infection of mice with *P. gingivalis* and *F. nucleatum* is better than single infection models of experimental periodontitis [35]. Therefore, both *F. nucleatum* and *P. gingivalis* were used in the mouse model. In this pilot study, C57BL/6 J mice were randomly divided into three groups. Group I was untreated and served as sham-control Figure 5A. Group II and III were administered 4NQO for 8 weeks in their drinking water. Group III mice were infected with *P. gingivalis* and *F. nucleatum* orally 3 times per week as shown in Figure 5A. During the period of 4NQO administration, mice were not infected with bacteria. We used combined infection of *P. gingivalis* and *F. nucleatum* to simulate human infections where *F. nucleatum* do not present alone in periodontal infections.

Histopathological analysis of tongue tissue sections indicated that Group III mice showed markedly enhanced hyperplasia and increased nuclear/cytoplasmic ratio compared to Group I and II Figure 5(B,C). Group III also had more oral lesions compared to Group II Figure 5D. Furthermore, lesions in Group III were significantly larger in size than Group II. In general, there was a significant increase in the lesion area when the bacteria were orally infected along with 4NQO Figure 5E. No lesions were detected in Group I.

## Discussion

For many years, chronic inflammation has been linked to the initiation and progression of cancer. Chronic inflammation in periodontal disease leads to destruction of the bone surrounding the teeth [36]. It has been suggested that prolonged exposure to periodontal bacteria and inflammation could increase the risk of oral cancer [7]. Epidemiological studies have supported this association. Periodontitis was associated with higher risk of oral cancer in a cross-sectional study using the Third National Health and Nutrition Examination Survey [9]. Another study concluded that patients with periodontitis were more likely to have poorly differentiated OSCC than those without periodontitis [37]. Periodontal bacteria exist in and around cancer lesions [38,39]. Hence, the present study was to investigate the effect of periodontal bacteria on oral cancer cell behaviour.

The observation that *Fusobacteria* upregulated STAT3, JAK1, and MYC indicated that *Fusobacteria* infection might enhance cancer cell survival through modulation of these oncogenes, which are well implicated in pathways that promote tumorigenesis [24]. Enhanced IL-8 secretion by *Fusobacteria* is not only associated with promoting cancer cell invasion, but also found in the saliva of oral cancer patients and implicated in disease progression [32,40]. The presented data together support the concept that *F. nucleatum* contributes to oral cancer survival and invasiveness.

*F. nucleatum* is a gram-negative anaerobic bacterium that was first isolated from the oral cavity and identified as a periodontal pathogen. Recent studies have reported *F. nucleatum* detected in different infection sites, including endocarditis, septic arthritis, and liver and brain abscesses. *F. nucleatum* is implicated in adverse pregnancy outcome [41]. *F. nucleatum* has also been isolated from colon cancer lesions and proposed to have a role in cancer progression and immune system modulation [42–44]. In colon cancer, *F. nucleatum* is linked to chemoresistance and associated with distant metastasis [16,45]. *F. nucleatum* enhanced colorectal cancer cell proliferation by activation of NF-κB and upregulation of miR-21 [46]. Moreover, FadA protein on *F. nucleatum* can bind to E-cadherin on colorectal cancer cells, leading to activation of the potentially oncogenic E-cadherin–β catenin signalling pathway [26]. *F. nucleatum* has also been shown to promote cell proliferation and migration in epithelial cells through up-regulation of cell cycle kinases. In oesophagal cancer, cancerous tissues contained significantly more *F. nucleatum* DNA than matched normal oesophagal mucosa, and increased levels were associated with poor prognosis [47]. Overall, *Fusobacteria* have been linked to carcinogenesis in multiple cancer types and appear to impact cancer progression and treatment response. However, there are still many gaps to be filled in understanding the biological role of *Fusobacteria* in enhancing oral cancer progression.

To investigate the mechanism by which *Fusobacteria* could have such an effect on cancer cells, we infected oral cancer cells either with live bacteria, heat-killed bacteria, or bacterial supernatant. In addition to increased secretion of IL-8 and MMPs, OQ01 cells infected by live bacteria clearly induced substantially higher expression of STAT3, MYC, and ZEB1 than those treated with heat-killed bacteria or bacterial supernatant alone. The reduction in IL-8 secretion by PMB treatment indicated that LPS was the bacterial supernatant component largely responsible for enhancing IL-8 secretion. Interestingly, *Fusobacteria* culture supernatant alone was sufficient to induce IL-8 and MMP expression, suggesting that *Fusobacteria* secretes an active compound(s) that could promote carcinogenesis without direct contact of the bacteria with tumour cells. Further investigation revealed that *Fusobacteria* might interact with oral cancer cells through multiple pathways. One of which was the LPS/TLR4 pathway that induced IL-8 secretion in OQ01 cells. However, the same pathway did not affect MMP1 and MMP9 expression suggesting the role of a second pathway in enhancing MMPs expression. Our results are consistent with the data published on *Fusobacteria*/colon cancer cells interaction showing that *Fusobacteria* can activate multiple pathways in cancer cells, including E-cadherin and NF-KB pathways [26,46].

The results observed in this study showed some variability in response among the three cancer cell lines examined. This can be attributed to several factors, including the heterogeneity (different mutations) among different cancer cell lines make their response to bacterial infection variable and the potential difference in their origins in the oral cavity may account for difference in their interaction with bacteria.

The role of periodontal bacteria in oral carcinogenesis was followed up in the 4NQO carcinogenesis model. 4NQO is a carcinogen known to induce DNA damage, leading to early premalignant and malignant lesions in the oral cavity [48]. Our data showed clearly that combined repeated infections with *P. gingivalis* and *F. nucleatum* induced twice as many lesions compared to exposure to 4NQO alone. The sizes of lesions were ~3 times larger in bacteria-infected versus non-infected 4NQO-treated mice. Our data is consistent with results reported by Binder Gallimidi et al. that infection with *P. gingivalis* and *F. nucleatum* promote tumorigenesis in their 4NQO carcinogenesis mouse model [33]. Future studies should examine whether both bacteria are required and their relative contributions to the promotion and severity of oral lesions.

In summary, our *in vitro* and *in vivo* studies provide evidence for the role of *Fusobacteria* in oral cancer progression and invasion. Relevant to our observations is the recent metatranscriptomic study that reported significantly higher transcripts of *Fusobacteria* at oral tumour sites and adjacent sites compared to the healthy controls analyzed [14]. Understanding the role of periodontal infections in cancer progression is important, as it may provide new strategies to improve treatment outcomes.

## Supplementary Material

Supplemental Material
