# Supplementary material for: Fusobacteria modulate oral carcinogenesis and promote cancer progression
Source: J Oral Microbiol. 2020 Nov 30;13(1):1849493. doi: 10.1080/20002297.2020.1849493 (PMC7717872; doi:10.1080/20002297.2020.1849493)
Supplement: Supplemental Material [file ZJOM_A_1849493_SM6413.pdf]

Fusobacteria modulate oral carcinogenesis and promote cancer progression

Amani M. Harrandah, Sasanka S. Chukkapalli, Indraneel Bhattacharyya, Ann Progulske-Fox, and Edward K. L. Chan

Appendix

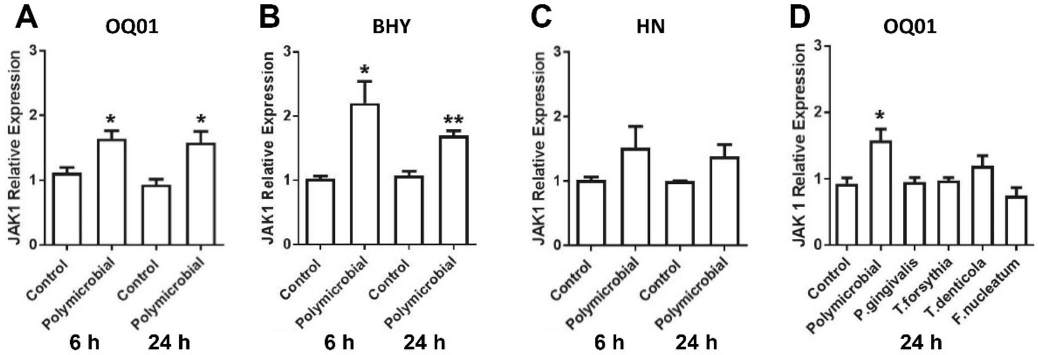

Appendix Figure 1. Moderately elevated JAK1 mRNA expression detected after polymicrobial infection. OQ01 (A), BHY (B), and HN (C) cells were infected with a mixture of four bacteria for 6 and 24 h as described in Figure 1A. Single bacterial infections in OQ01 cells showed no significant increase in JAK1 mRNA expression 24 h after infection (D). All mRNA levels were determined using qRT-PCR. All results are presented as mean ± SEM from three independent experiments. Statistical significance was determined by two-tailed Student's t test comparing to control uninfected cells (*, P < .05; **, P < .005).

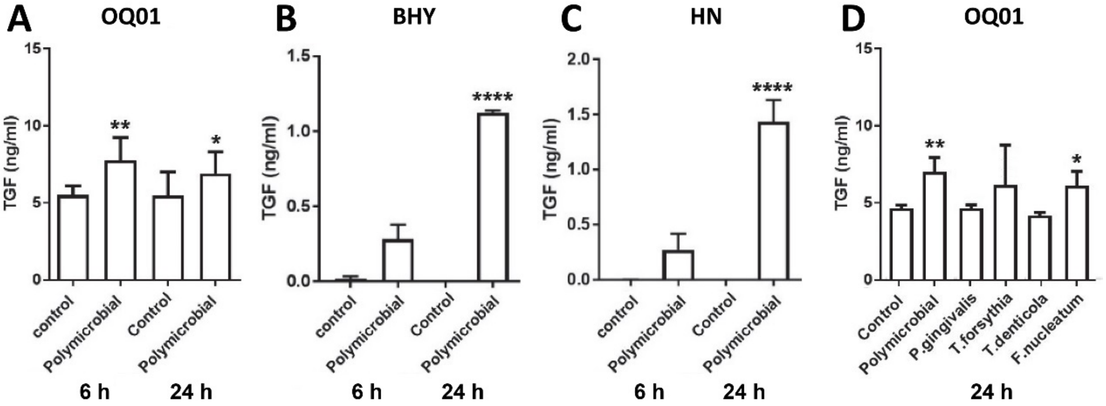

Appendix Figure 2. *F. nucleatum* promotes expression of epithelial-mesenchymal transition (EMT) marker TGF-β in oral cancer cells. (A-C) Increased secretion of TGF-β protein in OQ01, BHY, and HN cell supernatant after polymicrobial infection for 6 and 24 h as determined by ELISA. (D) Comparison of the effects of polymicrobial and single infections on TGF-β secretion in OQ01 cell supernatant determined by ELISA. For all experiments, uninfected cells were used as control. All results are presented as mean ± SEM from three independent experiments. Statistical significance was determined by two-tailed Student's t test comparing to control uninfected cells (*, P < .05; **, P < .005; ****, P < .0001).

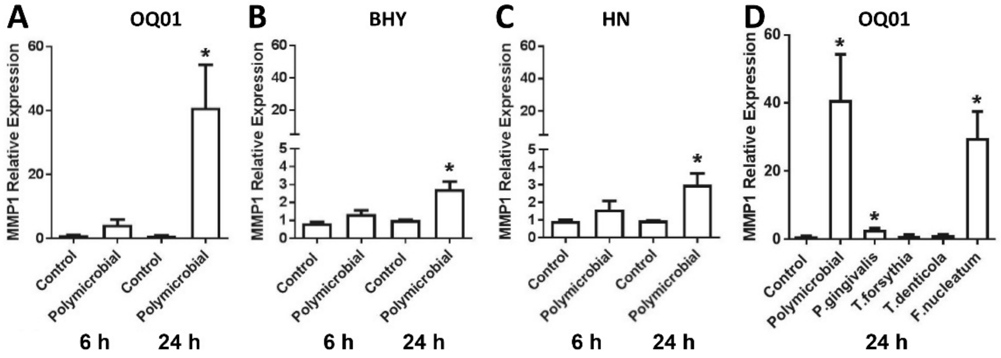

Appendix Figure 3. *F. nucleatum* enhances MMP1 expression. (A-C) MMP1 mRNA expression levels after polymicrobial infection of OQ01, BHY, and HN, respectively, for 6 and 24 h. (D) Differences in MMP1 expression levels in OQ01 cells after single infection with each of the four bacteria. All mRNA levels were determined using qRT-PCR. Uninfected cells were used as controls. All results are presented as mean ± SEM from three independent experiments. Statistical significance was determined by two-tailed Student's t test comparing to control uninfected cells (*, P < .05).
